# Nordic survey on assessment and treatment of fluid overload in intensive care

**DOI:** 10.3389/fmed.2022.1067162

**Published:** 2022-11-25

**Authors:** Emilie Zeuthen, Sine Wichmann, Martin Schønemann-Lund, Mikko J. Järvisalo, Rebecka Rubenson-Wahlin, Martin I. Sigurðsson, Erling Holen, Morten H. Bestle

**Affiliations:** ^1^Department of Anesthesia and Intensive Care, Copenhagen University Hospital, North Zealand, Denmark; ^2^Perioperative Services, Intensive Care and Pain Medicine, Turku University Hospital, Turku, Finland; ^3^Kidney Center, Turku University Hospital, University of Turku, Turku, Finland; ^4^Department of Clinical Science and Education, Södersjukhuset, Karolinska Institutet, Stockholm, Sweden; ^5^Department of Anesthesia and Intensive Care, Södersjukhuset, Stockholm, Sweden; ^6^Department of Anesthesia and Critical Care, Landspitali – The National University Hospital of Iceland, Reykjavík, Iceland; ^7^Faculty of Medicine, University of Iceland, Reykjavík, Iceland; ^8^Department of Anesthesia and Intensive Care, Helse Stavanger University Hospital, Stavanger, Norway; ^9^Department of Clinical Medicine, University of Copenhagen, Copenhagen, Denmark

**Keywords:** fluid accumulation, fluid removal, survey, diuretics, fluid overload, ICU, de-resuscitation

## Abstract

**Introduction:**

Fluid overload in patients in the intensive care unit (ICU) is associated with higher mortality. There are few randomized controlled trials to guide physicians in treating patients with fluid overload in the ICU, and no guidelines exist. We aimed to elucidate how ICU physicians from Nordic countries define, assess, and treat fluid overload in the ICU.

**Materials and methods:**

We developed an online questionnaire with 18 questions. The questions were pre-tested and revised by specialists in intensive care medicine. Through a network of national coordinators. The survey was distributed to a wide range of Nordic ICU physicians. The distribution started on January 5th, 2022 and ended on May 6th, 2022.

**Results:**

We received a total of 1,066 responses from Denmark, Norway, Finland, Sweden, and Iceland. When assessing fluid status, respondents applied clinical parameters such as clinical examination findings, cumulative fluid balance, body weight, and urine output more frequently than cardiac/lung ultrasound, radiological appearances, and cardiac output monitoring. A large proportion of the respondents agreed that a 5% increase or more in body weight from baseline supported the diagnosis of fluid overload. The preferred de-resuscitation strategy was diuretics (91%), followed by minimization of maintenance (76%) and resuscitation fluids (71%). The majority declared that despite mild hypotension, mild hypernatremia, and ongoing vasopressor, they would not withhold treatment of fluid overload and would continue diuretics. The respondents were divided when it came to treating fluid overload with loop diuretics in patients receiving noradrenaline. Around 1% would not administer noradrenaline and diuretics simultaneously and 35% did not have a fixed upper limit for the dosage. The remaining respondents 63% reported different upper limits of noradrenaline infusion (0.05–0.50 mcg/kg/min) when administering loop diuretics.

**Conclusion:**

Self-reported practices among Nordic ICU physicians when assessing, diagnosing, and treating fluid overload reveals variability in the practice. A 5% increase in body weight was considered a minimum to support the diagnosis of fluid overload. Clinical examination findings were preferred for assessing, diagnosing and treating fluid overload, and diuretics were the preferred treatment modality.

## Introduction

Fluid overload is common in intensive care unit (ICU) patients and is associated with mortality in patients with sepsis (1), acute kidney injury (AKI) (2, 3), respiratory failure (4), traumatic brain injury (5), burn patients (6), and surgical patients (7). A previous systematic review found that the risk of mortality increased by 19% per one-liter increase in positive fluid balance on day 3 after admission to the ICU (8). Fluid overload reduces the functional reserve of the respiratory, gastrointestinal, nervous, and cardiovascular systems, as well as the liver and the kidneys (9).

Fluid overload during critical illness is hypothesized to be caused by a pathological increase in global vaso-permeability in combination with excessive intravenous administration of fluids (8). When diagnosing fluid overload, the cut-off definition of fluid overload in the field varies broadly (10, 11), and standardized markers for fluid balance are lacking. Thus, the diagnosis relies on surrogate parameters such as changes in body weight, cumulative fluid balance, clinical findings (such as edema), and radiological or ultrasound findings (9).

Loop diuretics are commonly applied to treat fluid overload in the ICU (12), but no solid evidence exists for this practice (13). Different strategies for minimization of fluid overload have been investigated. In this effort the term “de-resuscitation” was coined, covering several fluid removal strategies such as restricting resuscitation and maintenance fluids, using diuretics, or renal replacement therapy (RRT) (10). A systematic review summarized various de-resuscitation strategies and compared them to the standard of care or a liberal fluid strategy and found that de-resuscitation was associated with an increase in ventilator-free days, shorter stay at the ICU in patients with ARDS, sepsis, and SIRS (14). The review, however, found no reduction in mortality (14).

The clinical practice of de-resuscitation has been surveyed twice among ICU physicians (15, 16). One survey distributed to physicians in New Zealand (NZ), Australia (A), and The United Kingdom (UK) generated 219 responses (15). The respondents had a strong preference for RRT over diuretics when treating fluid overload in the oliguric/anuric patient and if the patient had a significant degree of fluid overload. Another survey was distributed to physicians through the United Kingdom Intensive Care Society, the European Society of Intensive Care Medicine, and subscribers to the mailing list from Criticalcarereviews.com (16). They received 524 responses, and most of the respondents were from the United Kingdom. The respondents preferred diuretics as a de-resuscitative strategy. Both surveys examined how physicians assess fluid status in ICU patients and both studies demonstrated how clinical examination findings, radiological findings, and changes in weight and fluid balance were the most frequent assessment modalities (15, 16).

A Nordic survey has, to our knowledge, never been conducted on this subject. We aimed to ascertain how Nordic ICU physicians define, assess, and treat fluid overload.

## Materials and methods

### Survey development

We developed a cross-sectional survey based on two previous survey articles (15, 16). We pre-tested these questions for face and construct validity (17). The pre-testing consisted of four semi-structured interviews with four ICU specialists, a pilot test, and a clinical sensibility test (Supplementary material 1) with six ICU physicians. The questionnaire was revised and modified according to the pre-tests. The survey was prepared according to the Consensus-Based Checklist for Reporting of Survey studies (CROSS checklist) (18). The Danish National committee on health research ethics (H-21064485) approved the study and the study was reported to The Capital Region Knowledge Centre for Data Compliance.

### Questionnaire

The final questionnaire contained 18 questions (Supplementary material 2). It contained multiple choice and Likert scale questions. Questions were demographical, attitudinal, and practice based. Participation was voluntary, and anonymous, and no personal data was collected. The survey data were collected and managed using Research Electronic Data Capture (REDCap) a secure, web-based software platform designed to support data capture for research studies (19).

### Survey distribution

The study population consisted of Nordic ICU physicians from Denmark, Sweden, Norway, Finland, and Iceland. We used purposive sampling by distributing through five national investigators, who invited local ICUs to distribute the survey link to their physicians through email invites. To manage the response rate the investigators filled out a distribution form for every department (Supplementary material 3). Reminder emails were sent either one or two times. The distribution started on January 5th, 2022 and ended on May 6th, 2022.

### Statistical analysis

We performed the statistical analyses with R, version 3.6.1 (20). In the analyses, we grouped the answer possibilities “agree/strongly agree,” “disagree/strongly disagreed,” “often/very often,” and “rarely/infrequently” when analyzing the results. Missing data were handled by case-wise deletion. We calculated the response rate according to the American Association of Public Opinion Research’s fourth definition of response rate (RR4) (Supplementary material 4) (21). Non-response bias was estimated using wave analysis (Supplementary material 5) (22).

## Results

### Respondent characteristics

**FIGURE 1:**
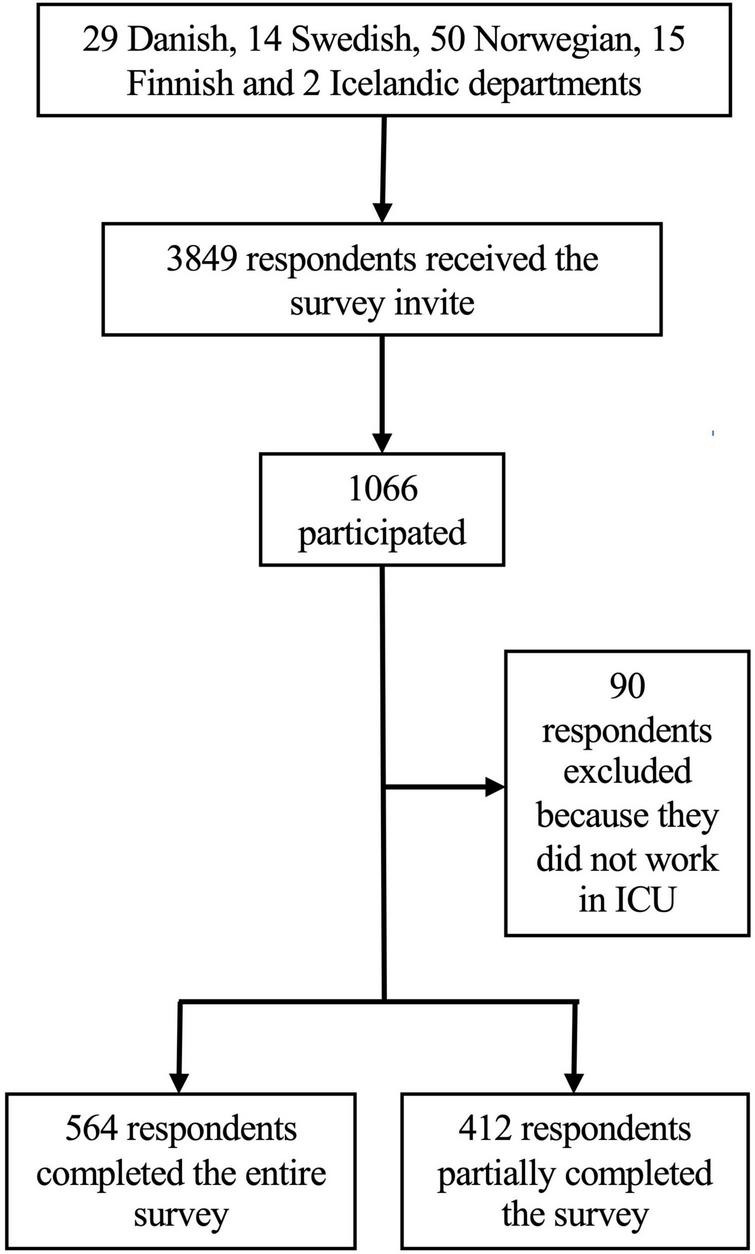
Flowchart over survey participation.

The survey was distributed to 3,849 physicians through 110 ICUs. The survey link was opened by 1,066 respondents, 90 respondents were excluded because their employment was not in the ICU, 564 respondents completed the full questionnaire, and 412 respondents partially completed the questionnaire (Figure 1). The majority (46%, 442/967) of the respondents were from Denmark, 20% (192/967) from Norway, 17% (168/967) from Sweden, 14% (133/967) from Finland, and 3% (32/967) from Iceland. Most physicians (82%, 789/967) worked in a mixed ICU, 41% (394/968) had more than 10 years of experience, and 64% (618/968) had a post-graduate qualification as a specialist. The response rate was 28% (Supplementary material 4) and the non-response bias was between −0.06 and 0.26 on a 5-point Likert scale (Supplementary material 5).

### Attitudinal questions about fluid overload

**FIGURE 2:**
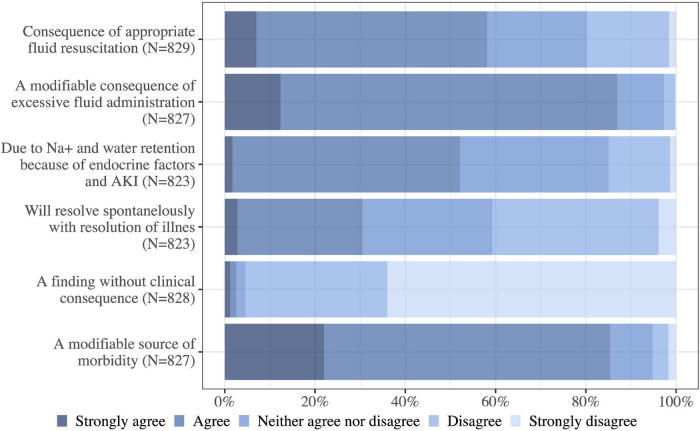
Broadly speaking how much do you agree with the following statements about the issue of fluid overload (positive fluid balance with edema) in ICU patients? Na, sodium; N, number of respondents to the question.

The majority of the physicians (85%, 816/957) reported fluid overload to be a common occurrence in their ICU. Most of the physicians (87%, 719/827) agreed/strongly agreed that fluid overload is a modifiable consequence of fluid administration and 86% (706/827) found it to be a modifiable source of morbidity. However, a majority also thought of fluid overload as an inevitable consequence of appropriate fluid resuscitation 58%, (482/829) agreed/strongly agreed and a manifestation of sodium and water retention due to endocrine factors and AKI (52%, 429/823 agreed/strongly agreed). The physicians were divided regarding the subject of fluid overload being an issue that resolves spontaneously, 31% (251/823) agreed/strongly agreed and 41% (335/823) disagree/strongly disagreed. The study demonstrated a broad consensus against fluid overload being a finding without clinical consequence (95% strongly disagreed/disagreed, 790/828) (Figure 2).

### Assessment of fluid overload

**FIGURE 3:**
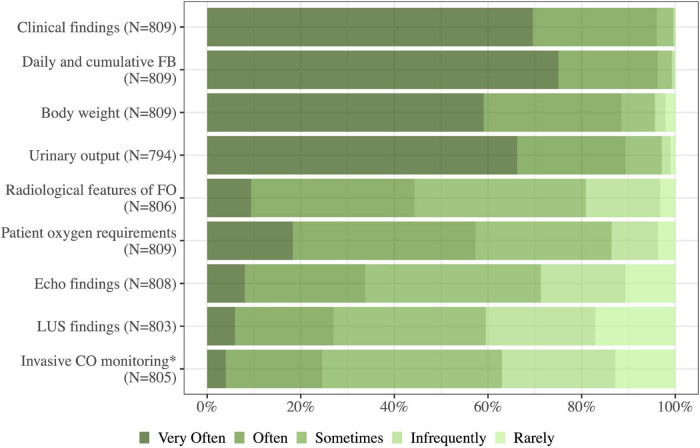
As a part of my routine clinical practice, I use the following when assessing the fluid status of a critically ill patient: FB, fluid balance; FO, fluid overload; LUS, lung ultrasound; CO, cardiac output; N, number of respondents to the question. *Results from invasive cardiac monitoring and measures of fluid responsiveness from these devices.

When assessing fluid status, physicians preferred clinical parameters. The physicians often or very often used clinical examinations such as the daily and cumulative fluid balance (96%, 779/809), edema (96%, 777/809), urinary output (89%, 710/794), body weight (88%, 716/809), and the patient’s oxygen requirements (57%, 464/809). Physicians used other modalities such as ultrasound and radiological appearances more sparsely (Figure 3).

### Diagnosing fluid overload

**FIGURE 4:**
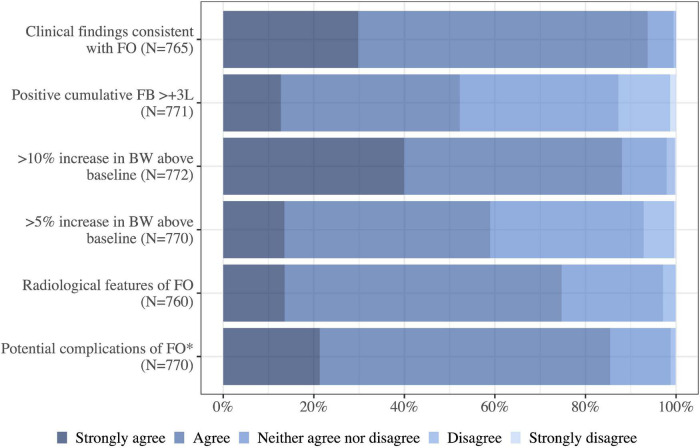
I agree that the following features support the diagnosis of fluid overload in a critically ill patient. FB, fluid balance; FO, fluid overload; BW, body weight; N, number of respondents to the question.*Presence of potential complications of fluid overload, e.g., increasing oxygen requirements, difficulty weaning from invasive ventilation, intraabdominal hypertension.

The physicians considered findings from clinical examination supportive of the diagnosis of fluid overload (94%, 717/765 agreed/strongly agreed). Additionally, when diagnosing fluid overload, 85% (658/770) found that the potential complications of fluid overload (increasing oxygen requirements, intra-abdominal hypertension, etc.), and radiological findings (75%, 568/760) were more supportive of the diagnosis of fluid overload than a positive cumulative fluid balance of 3 liters (52%, 403/771). Many of the respondents (88%, 680/772) agreed/strongly agreed to a 10% or more increase in body weight from baseline supported the diagnosis of fluid overload while 59% (454/770) of the physicians agreed to a minimum of 5% increase to support the diagnosis (Figure 4).

### Indications for de-resuscitation

**FIGURE 5:**
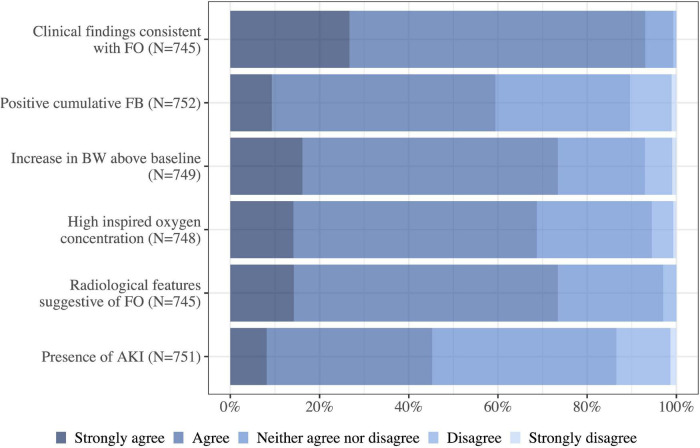
How much do you agree to each of the following indications for commencing de-resuscitation in the critically ill patient without shock? FB, fluid balance; FO, fluid overload; BW, body weight; N, number of respondents to the question; AKI, acute kidney injury.

Most physicians agreed/strongly agreed to clinical findings suggestive of fluid overload (93%, 693/745), radiological features suggestive of fluid overload (73%, 547/745) and increasing body weight (73%, 550/749) as indications for de-resuscitation. High inspired oxygen concentration (69%, 514/748) and a positive fluid balance (59%, 447/752) were additionally seen as indications. A total of 45% (340/751) agreed that AKI was an indication for commencing de-resuscitation (Figure 5).

### Management of fluid overload

**FIGURE 6:**
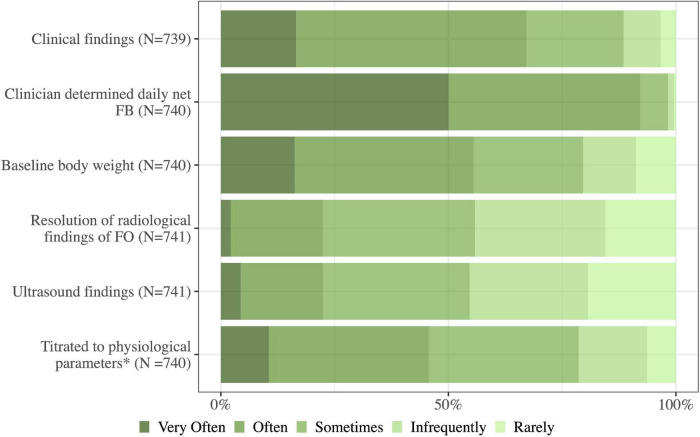
How often do you use the following approaches in the management of fluid overload in your daily practice? Fluid removal targeted to: FO, fluid overload; FB, fluid balance; N, number of respondents to the question. *Fluid removal titrated to physiological parameters (cardiac output measurements, blood pressure, and gas exchange).

The ICU physicians preferred fluid removal targeted to daily net fluid balance (92%, 682/740 would use these modalities often/very often) followed by clinical examination findings (67%, 496/739) and baseline body weight (56%, 411/740). Other modalities such as ultrasound findings or physiological parameters to target fluid removal were rarely used (Figure 6).

### Treatment of fluid overload

**FIGURE 7:**
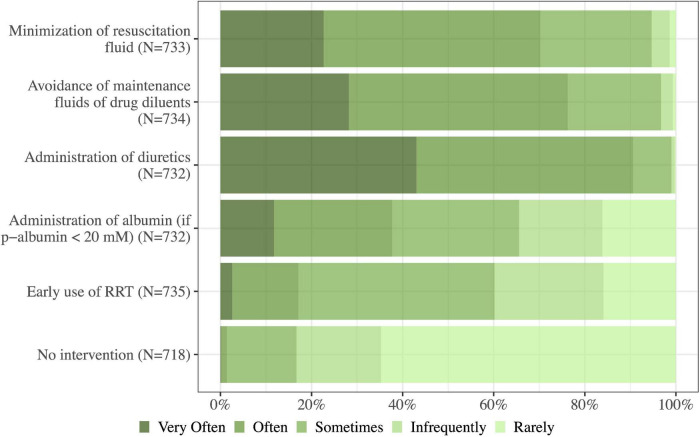
How often do you use the following strategies to avoid or deal with fluid overload in the critically ill? RRT, renal replacement therapy; N, number of respondents to the question.

**FIGURE 8:**
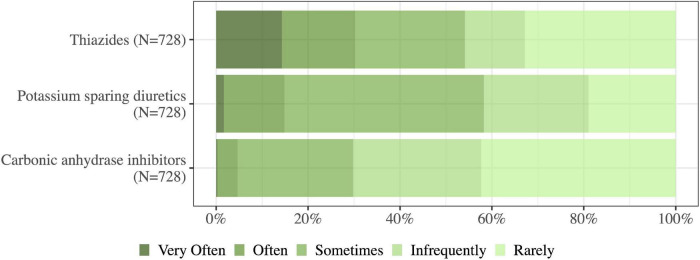
Of the occasions on which you administer loop diuretics to achieve a negative fluid balance, how often do you use the following agents (either as adjuncts or alternatives)? N, number of respondents to the question.

Diuretics were the preferred de-resuscitation strategy (91%, 663/732 of physicians used these often/very often) followed by minimization of maintenance fluids and drug diluents (76%, 560/734) and minimization of resuscitation fluids (70%, 515/733). Administration of albumin was used sometimes/often or very often by 66% (480/732) of the physicians in case of in case of plasma albumin < 20 mM. Commencement of RRT was less likely, but 83% (598/718) of physicians would rarely/infrequently leave an ICU patient with fluid overload untreated (Figure 7). When choosing an adjunct or an alternative diuretic to loop diuretics potassium-sparing diuretics and thiazides were preferred over carbonic anhydrase inhibitors (Figure 8).

### Revision of the de-resuscitative strategy

**FIGURE 9:**
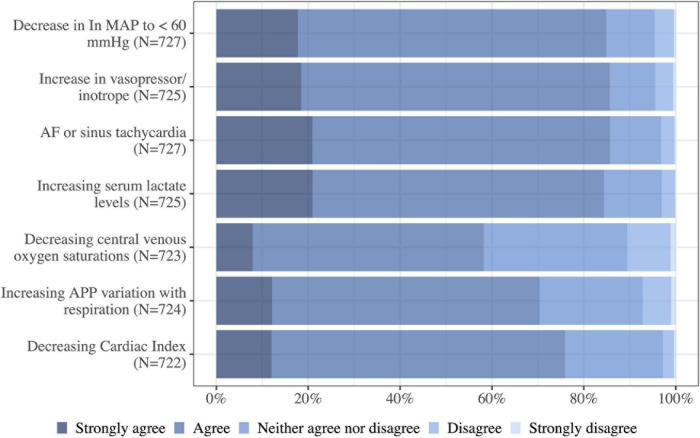
Do you agree that the following situations would trigger a review of the planned rate of fluid removal in a critically ill patient? MAP, mean arterial pressure; AF, atrial fibrillation; N, number of respondents to the question.

A majority agreed/strongly agreed that they would review their fluid removal plan in all the listed clinical situations in Figure 9.

### Contraindications and adverse effects of diuretics

**FIGURE 10:**
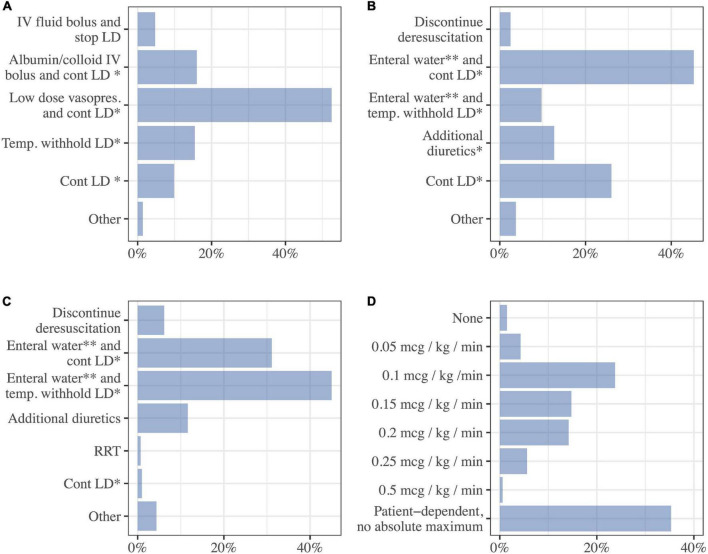
Complications and contraindications. Answer the following questions while you assume you are attending a stable critically ill patient with fluid overload, who is treated with loop diuretics to achieve a negative fluid balance. Choose the option that best describe your likely response. **(A)** Mild hypotension (MAP 55–65 mmHg) (*N* = 718), **(B)** mild hypernatremia (145–150 mmol/L) (*N* = 718), **(C)** severe hypernatremia (>150 mmol/L) (*N* = 713), **(D)** max dose of noradrenaline that is acceptable while continuing using diuretics for fluid removal (*N* = 677). LD, loop diuretics; cont., continue; Vasopres, vasopressor; Temp, temporarily; RRT, renal replacement therapy; *Monitor closely, ^**^or 5% dextrose IV.

The physicians were questioned about how they would react to different complications and contraindications when having commenced loop diuretics in a stable ICU patient with fluid overload. In the case of mild hypotension (MAP 55–65 mmHg) the physicians were most likely to commence low-dose vasopressors, continue loop diuretics, and monitor closely (53%, 377/718) (Figure 10). In case of mild hypernatremia (145–150 mmol/l), the majority would administer enteral water and continue loop diuretics (45%, 325/718). If the hypernatremia was more severe (above 150 mmol/l) the majority would administer water and temporarily withhold loop diuretics (45%, 321/713) while 31% (222/713) of physicians would administer water and continue loop diuretics. The physicians were asked about the acceptable dose of noradrenaline infusion when administering diuretics. The respondents upper limit for noradrenaline dosage ranged between 0.05 and 0.5 mcg/kg/min while most respondents 52% (357/677) had an upper limit between 0.1 and 0.2 mcg/kg/min, a substantial part of the respondents 35% (239/677) had no fixed upper limit for noradrenaline infusion. A minority would not administer diuretics and noradrenaline simultaneously (1%, 10/677) (Figure 10).

## Discussion

We have conducted a Nordic survey on self-reported practices used in the ICU concerning patients with fluid overload. Our survey reveals variability in the practice, but some tendencies emerge. Clinical examination findings were the most preferred modality when assessing, diagnosing, and targeting fluid strategy. This is in line with previous surveys in the field that demonstrated how clinical examination findings, changes in body weight, and fluid balance were the most frequent assessment modalities in the ICU (15, 16). The most notable difference between the physicians surveyed from NZ, UK, and Australia compared to the Nordic physicians was the use of body weight. The UK, NZ, and Australians were less likely to target fluid removal to baseline body weight (11% in NZ/A/UK vs 56% in Nordic countries) and were less likely to use an increase in body weight as an indication to commence de-resuscitation (NZ/A/UK 45% vs Nordic countries 73%) (15).

Precise determination of fluid balance is known to be complicated. The Nordic physicians most commonly used clinical examination findings to assess fluid overload, this is potentially problematic for several reasons. The formation of peripheral and pulmonary edema is complex and formed due to many contributing factors in addition to fluid overload (9). Body weight might be a good indicator of fluid balance but is affected by other factors such as loss of muscle and fat mass during ICU stay (9). Fluid balance is difficult to chart precisely, is prone to calculation errors, and also includes several parameters, which can only be estimated such as stool, insensible water loss, and perspiration (23). At ICU admission, patients are often registered with a neutral fluid balance, but they might have fluid accumulation or be dehydrated ahead of admission without proper registration. A systematic review of 13 cohort studies found that in estimating fluid status in patients in the ICU, both fluid balance and body weight were imprecise, difficult to perform, and inconsistent with one another (23). This emphasizes a lack of scientific evidence for the most preferred fluid assessment modalities in the Nordic ICUs. In previous surveys physicians had similar preferences (15, 16) which demonstrates a potential limitation when assessing fluid overload in the ICU beyond the Nordic countries.

Radiological findings, echocardiograms, and lung ultrasound were used to a certain extent by the Nordic physicians when assessing fluid status. The survey from NZ/A/UK demonstrated different preferences concerning these modalities. Nordic physicians used ultrasound more frequently, especially lung ultrasound (27% of Nordic physicians vs. 8% of physicians from NZ/A/UK used it often/very often). Conversely, UK/A/NZ physicians used radiological findings more frequently (69% of NZ/A/UK physicians vs. 44% of Nordic physicians).

Chest X-ray findings can be difficult to interpret in ICU patients and the diagnostic performance for fluid overload is at best mediocre (24). Ultrasound examinations are interpreted subjectively, require technical expertise, and can be difficult to perform (9). However, one small observational study showed that the use of echocardiography and lung ultrasound in the ICU, altered fluid therapy in 25% of the patients, and the hemodynamic diagnosis was changed in 66% (25). This provides preliminary evidence that bedside ultrasound may be a valuable tool in the diagnosis and management of fluid overload in patients in the ICU, and new systematic protocols for Venous Excess Ultrasound Grading System (VEXUS) are currently being tested (26).

Multiple retrospective studies have shown that an increase above both 5 and 10% weight-based cumulative fluid balance is significantly correlated with increased mortality in AKI (27) and surgical patients (7). These findings were most predominant in the groups with fluid overload above 10% (7), but a systematic review found that the risk of mortality increased by 19% per liter increase in positive fluid balance (8). Many Nordic physicians diagnose fluid overload as an increase above 10% in body weight and a large part of the physicians would also diagnose fluid overload as an increase above 5%. This underlines how Nordic physicians are aware of the possible risks associated with fluid accumulation. Similarly, in the survey study from NZ, Australia and the UK 76% of the respondents found a 10% increase in body weight supported the diagnosis of fluid overload.

Acute kidney injury was considered an indication for de-resuscitation amongst a large portion of the Nordic physicians (45% out of 751). There might be a divide in the understanding of this question. Some respondents might interpret it the question as AKI either with or without fluid overload. Our intention was to inquire the indication for commencing de-resuscitation in the case of AKI and fluid overload. International guidelines from the Kidney disease: Improving global outcomes (KDIGO) suggests not using loop diuretics in the case of AKI except in the case of fluid overload (28). Given that the respondents understood the question as it was intended, would demonstrate how Nordic physicians are aware of current recommendations from KDIGO. If the question was understood as AKI without fluid overload the physicians would surprisingly disagree with the current recommendations.

The Nordic physicians were inclined to intervene in the case of fluid overload in their patients. Diuretics were the most preferred method for de-resuscitation followed by minimization of maintenance fluids, resuscitation fluids, and drug diluents. A previous international survey distributed among three intensive care societies found similar results (16). RRT was preferred more than diuretics when the patients were oliguric/anuric, had a significant fluid overload, and in the case of AKI even when the traditional indications for RRT were not present (15). Due to the sparse number of interventional studies in the field, systematic reviews on interventions in established fluid overload have pooled all de-resuscitative measures and also observational studies and interventional studies are pooled (8, 10, 14). Essential findings were an increase in ventilator-free days and a decrease in days in the ICU in the de-resuscitative and conservative groups (14). The largest systematic review on loop diuretics in ICU patients with fluid overload showed no difference in mortality in patients treated with loop diuretics versus placebo/no intervention (13).

Most studies in the field of fluid therapy focus on resuscitation fluids but also maintenance fluids and fluid creep (fluid volume administered unintentionally from enteral, oral, and intravenous medication) are substantial sources of fluid accumulation (29). In one study, maintenance fluids and fluid creep were responsible for 25% and 33% of all administered fluids, respectively while resuscitation fluids were only responsible for 7% (29).

The evidence for using a combination of different types of diuretics is sparce for the general ICU patient (30). The existing trials are small and with inconclusive results (30–33). Evidence supports diuretic combinations in the management of heart failure. It is recommended in both American and European Heart Failure Guidelines when the diuresis is inadequate with loop diuretic therapy thiazides may be considered in addition (34, 35). In our survey we asked which type of diuretics the ICU physician used as adjunct while administering loop diuretics. It was a general question not directed at subgroups of patients – the answers might have been different if we had addressed subgroups of patients as heart failure. The respondents primarily used thiazides and potassium-sparring diuretics and less frequently carbonic anhydrase inhibitors which is in line with former survey (16).

A large portion of the respondents recognize increasing oxygen requirements and radiological findings as indications for commencing de-resuscitation, presumably because these are surrogate markers for ARDS (36). Current guidelines from the Scandinavian Society of Anaesthesiology and Intensive Care Medicine (SSAI) on ARDS suggest a conservative fluid strategy but not diuretics specifically (37). This demonstrates how Nordic countries are in keeping with current guidelines concerning ARDS and fluid management (37).

A large portion (66%, 480//732) of the Nordic physicians were inclined to administrate intravenous albumin in the case of p-albumin less than 20 mM. Albumin is recommended in the case of ARDS in newer English guidelines (38) but not by SSAI (37). Our question addressed hypoalbuminea and fluid overload not ARDS specifically. It is being hypothesized that hypoalbuminea might cause diuretics resistance and should be co-administrated with furosemide (38). One meta-analysis examined the co-administration of albumin and furosemide on diuretic resistance. It demonstrated no reduction in all-cause mortality after 30 days, but found a reduction in hypotensive events and improved PaO_2_/FiO_2_ ratio at 24 h (39).

The respondents were divided about treating fluid overload with loop diuretics in patients receiving noradrenaline. Few physicians would not administrate noradrenaline and loop diuretics simultaneously. The majority of respondents 63% had a fixed upper limit of noradrenaline dose between 0.05 and 0.5 mcg/kg/min and 35% of respondents found it patient dependent and did not have an upper limit. Another survey in the field found that 50% of the physicians had no fixed upper, the physicians from that survey were thus more liberal than the Nordic physicians (16). This practice of using vasopressors and diuretics is supported by findings from a large observational study, which showed a decrease in mortality in patients with fluid overload who were on vasopressor support and treated with diuretics concurrently (40). This has not been tested in a prospective randomized trial to our knowledge.

### Strengths and limitations

This study has considerable strengths. The questions in our survey have previously been applied and pretested for validity and have been conducted on similar study populations. We performed our own pretesting, a clinical sensibility test, and an item reduction, and remodified the survey before distribution. Another strength is the number of respondents we received. The size of the participant population indicates a proportionate representation of the ICU physicians in Nordic countries and of their general views.

The limitations of this study are the absence of a reliability test. Missing data were handled by case-wise deletion, and imputation might have given more precise results. Respondents of the survey might have stronger opinions or greater interest in the subject compared to the non-respondents, which could increase the non-response bias. The survey had 412 item non-responders which are respondents that neglected to answer one or more questions. All in all, there is a risk of significant non-response and potentially non-response bias in our study. We applied wave analysis to assess non-response bias by comparing how initial respondents answer the questionnaire compared to late respondents (proxies to non-respondents). The difference is interpreted on a five-point Likert scale, where two adjacent points (e.g., 1 and 2) represent adjacent statements on the Likert scale e.g., “Strongly disagree” and “Disagree.” Non-response bias in the survey was estimated to be small between −0.06 and 0.26, which demonstrates similar attitudes in the two groups. It shows a low possibility of bias in the survey (Supplementary material 5).

The response rate in the current study was 28%. The distribution of the survey varied in the participating countries which is visible when comparing the percentage of physicians from each Nordic country. Our sampling strategy was relatively broad which could have resulted in distribution to physicians who might not work in the ICU and therefore were less likely to open the survey link. There can also be physicians who are employed in more than one ICU and potentially have received the survey invitation more than once. This demonstrates that the response rate might be skewed and would be more precise if the distribution had been more selective.

## Conclusion

Self-reported practices among Nordic ICU physicians when assessing, diagnosing, and treating fluid overload reveals variability in the practice. A 5% increase in body weight was considered a minimum to support the diagnosis of fluid overload. Clinical examination findings were preferred for assessing, diagnosing, and treating fluid overload, and diuretics were the preferred treatment modality.

## Data availability statement

The raw data supporting the conclusions of this article will be made available by the authors, at a reasonable request.

## Author contributions

SW and EZ designed the study. EZ and MS-L conducted the statistical analyses. All authors made a contribution to the acquisition of the data, manuscript, and approved the final version for publication.
