# Supplementary material for: Nordic survey on assessment and treatment of fluid overload in intensive care
Source: Front Med (Lausanne). 2022 Nov 25;9:1067162. doi: 10.3389/fmed.2022.1067162 (PMC9732460; doi:10.3389/fmed.2022.1067162)
Supplement: Supplementary file 1 [file Data_Sheet_1.docx]

**SUPPLEMENTARY MATERIAL**

This supplementary material has been provided by the authors to give readers additional information about their work.

Correspondence: Zeuthen E. Department of Anesthesia and Intensive Care, Copenhagen University Hospital − North Zealand, Denmarke-mail: [emilie.frederikke.zeuthen.norus@regionh.dk](mailto:emilie.frederikke.zeuthen.norus@regionh.dk)

**Content**

[**S1. Clinical sensibility testing tool From ESICM website (1)** 2](#_Toc116393483)

[**S2. Scandinavian survey on fluid overload in the ICU** 4](#_Toc116393484)

[**S3. Distribution survey to calculate response rate** 15](#_Toc116393485)

[**S4. Calculation of response rate:** 16](#_Toc116393486)

[**S5. Calculation of non-response bias** 17](#_Toc116393487)

[**S7. References** 21](#_Toc116393488)

## **S1. Clinical sensibility testing tool From ESICM website (1)**

1. To what extent are the questions directed at important issues pertaining the assessment and treatment of fluid overload in the critically ill?

| Small Extent | Limited Extent | Fair Extent | Moderate Extent | Large Extent |
| --- | --- | --- | --- | --- |
|  |  |  |  |  |

1. Are there other important elements regarding the assessment and treatment of fluid overload that should be included in the questionnaire which have been omitted?

| Crucial Gaps | Important Gaps | Minor Gaps | Minimal Gaps | Insignificant Gaps |
| --- | --- | --- | --- | --- |
|  |  |  |  |  |

Please identify any omissions: ______________________________________________________________

1. To what extent were the response options easy to understand?

| Small Extent | Limited Extent | Fair Extent | Moderate Extent | Large Extent |
| --- | --- | --- | --- | --- |
|  |  |  |  |  |

1. How many items you felt that the survey contained inappropriate or redundant questions?

| Very Many | Many | Some | A few | Hardly Any |
| --- | --- | --- | --- | --- |
|  |  |  |  |  |

1. How likely is that the survey will elicit response that can inform future research or inform practice

| Very Unlikely | Unlikely | Likely | Quite Likely | Very Likely |
| --- | --- | --- | --- | --- |
|  |  |  |  |  |

1. How long would it take you to complete the questionnaire? _________ minutes

## **
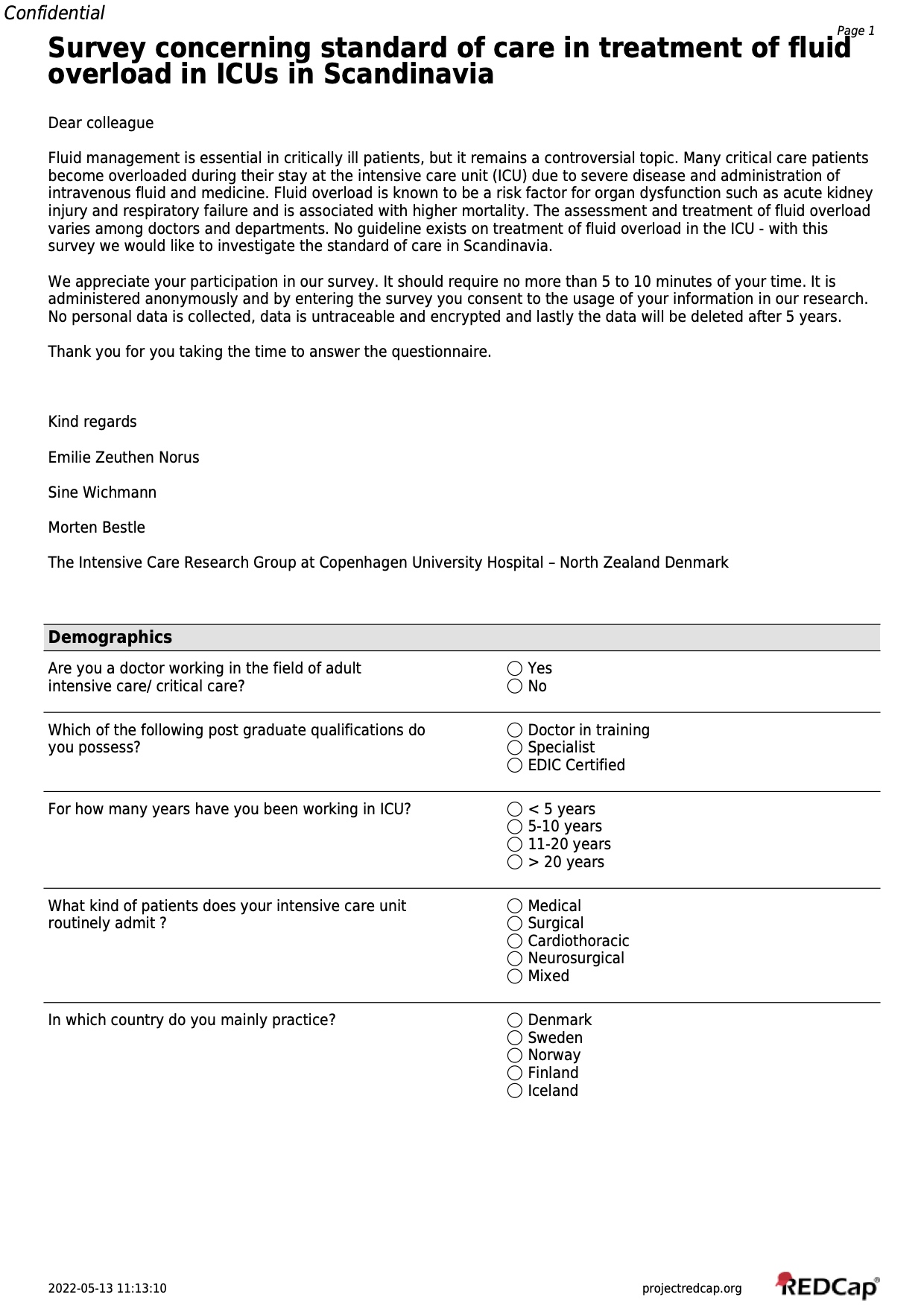
** **S2. Scandinavian survey on fluid overload in the ICU**

The survey is attached as screenshots of the PDF document


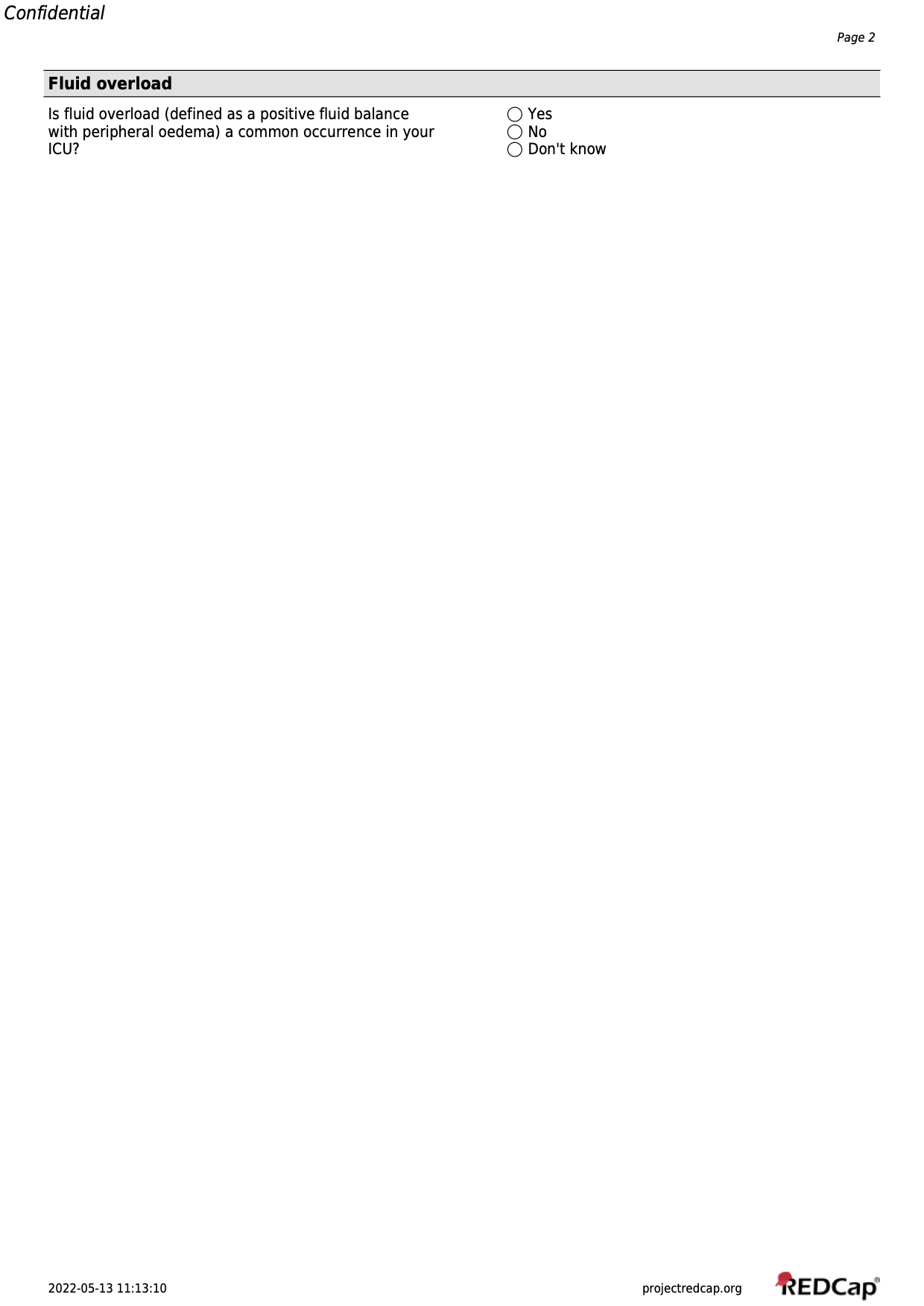


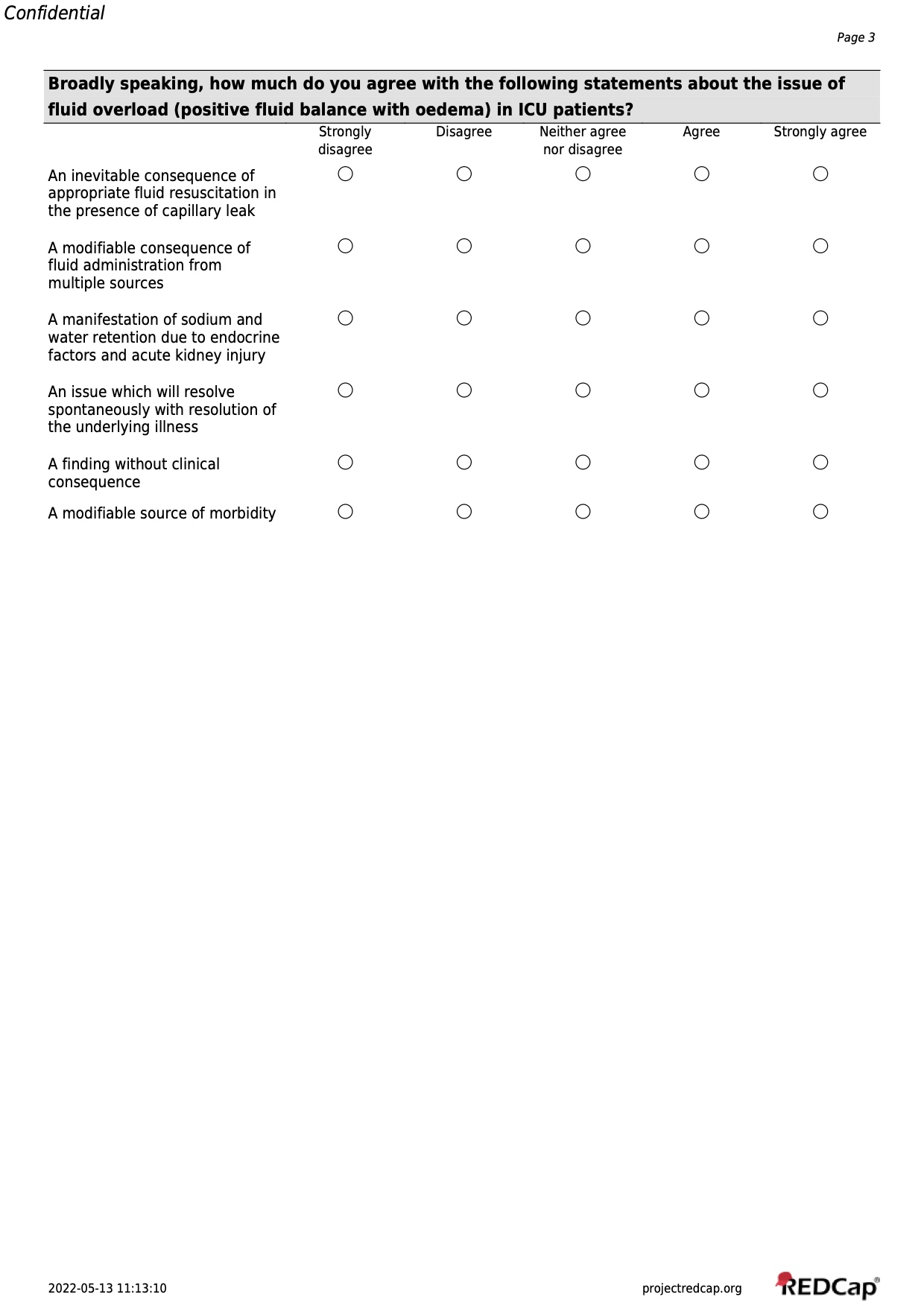


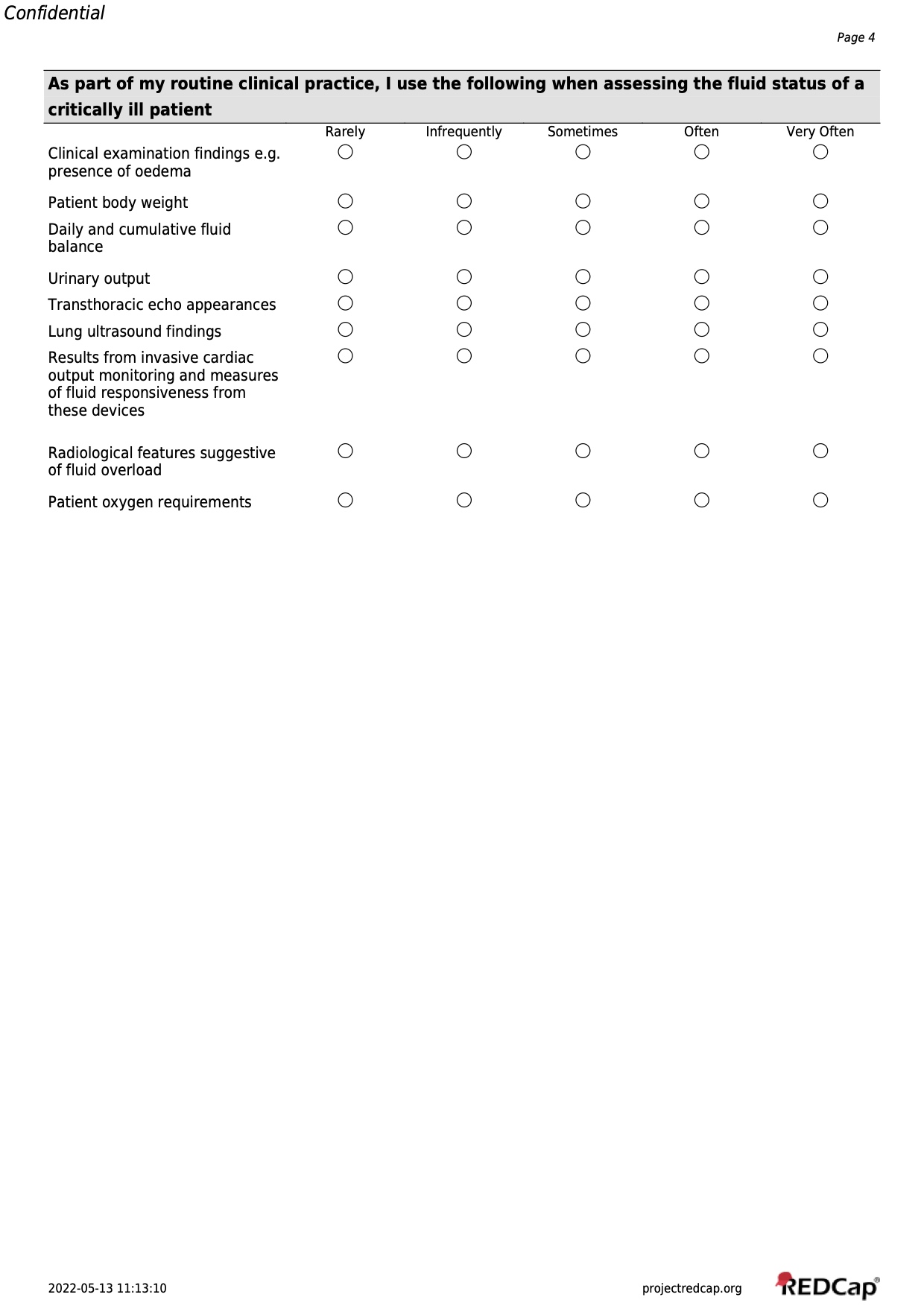


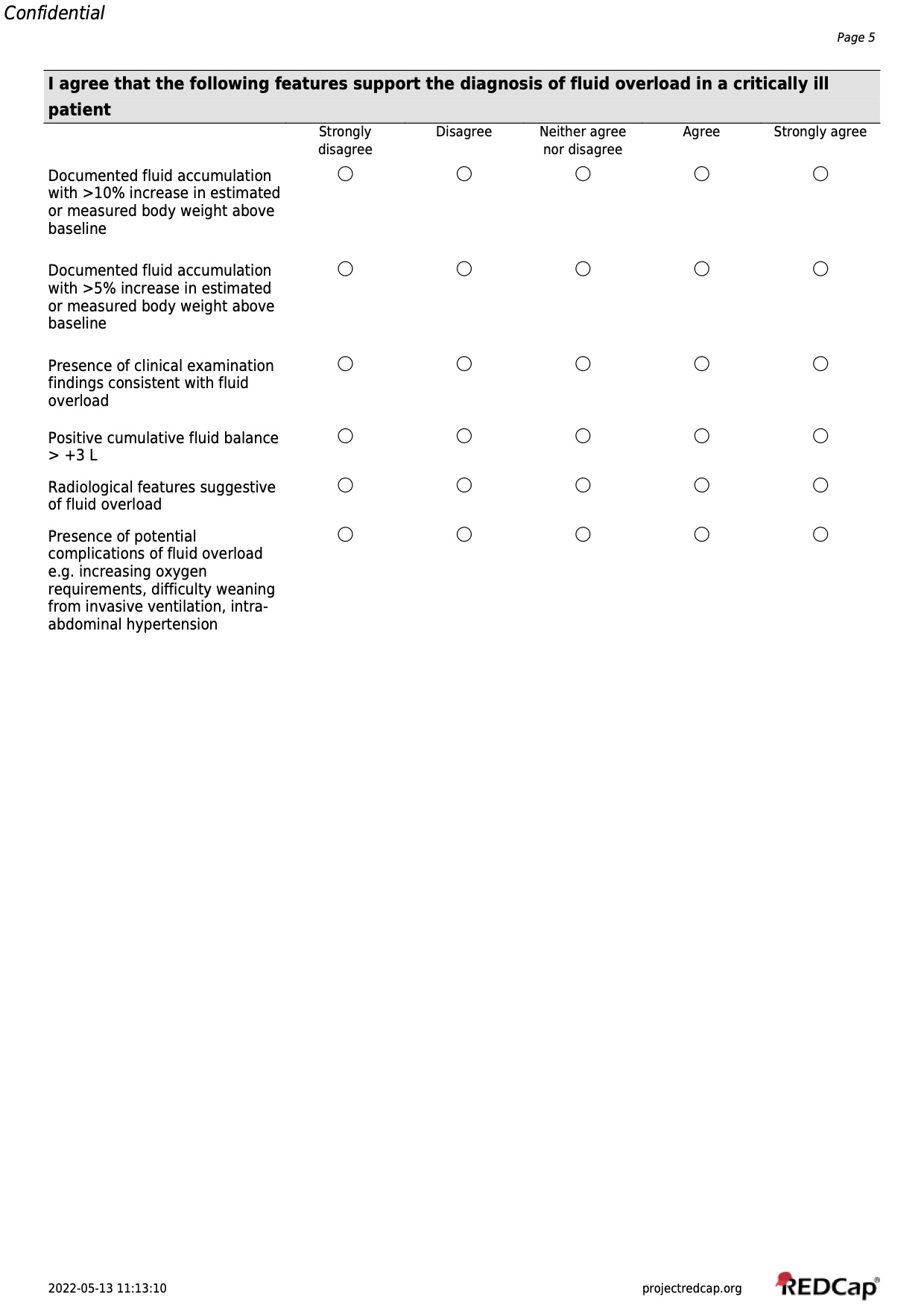

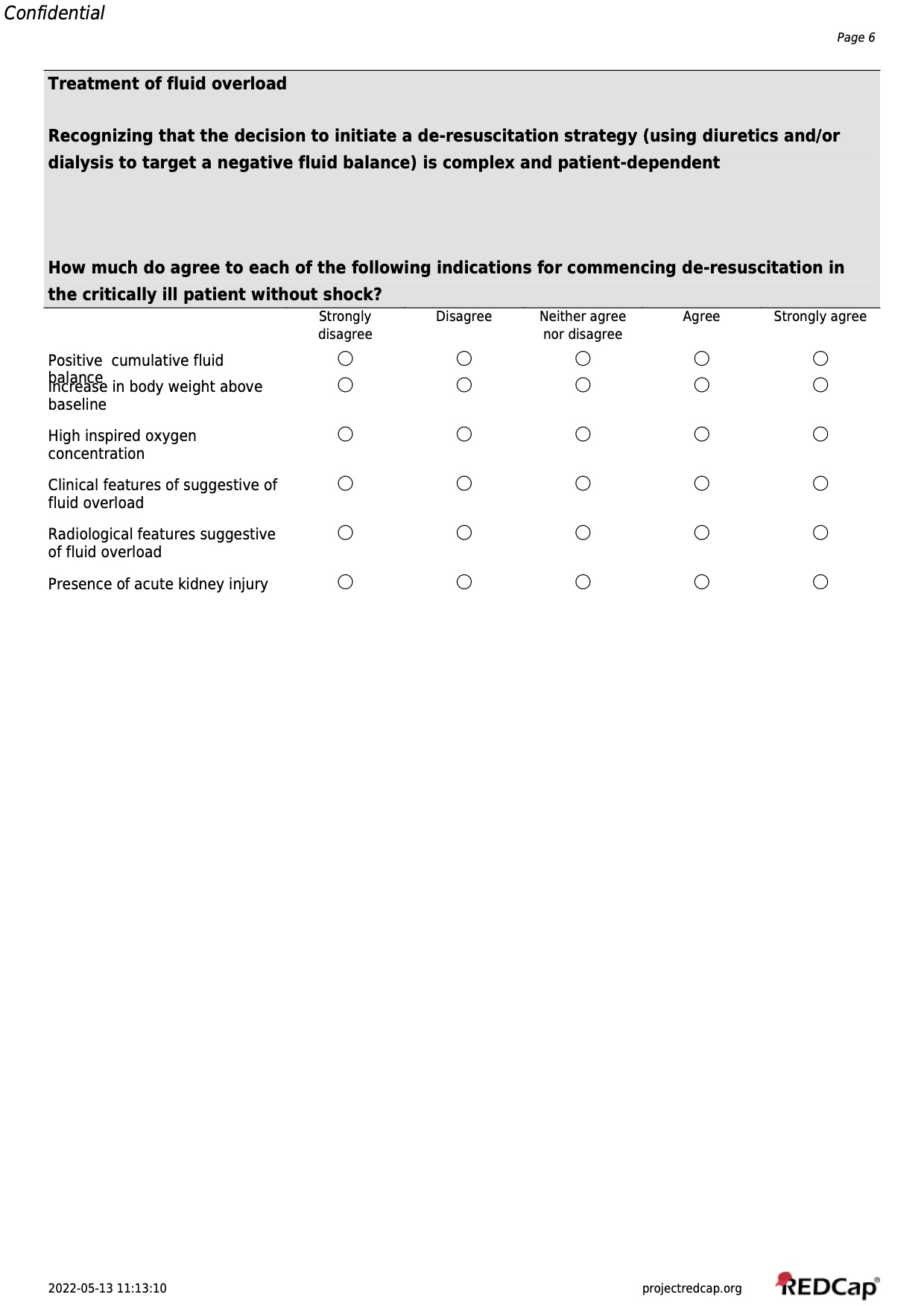

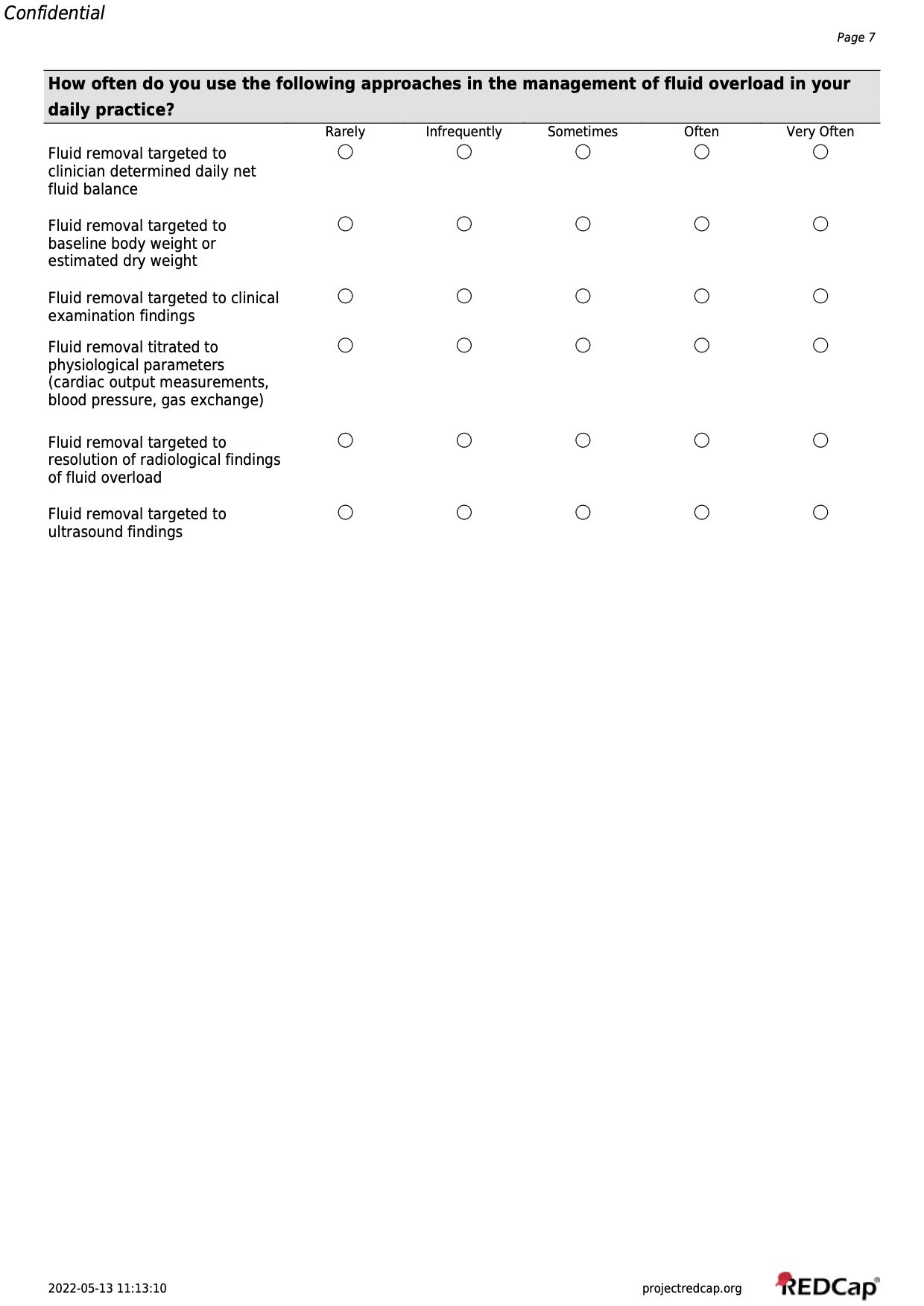

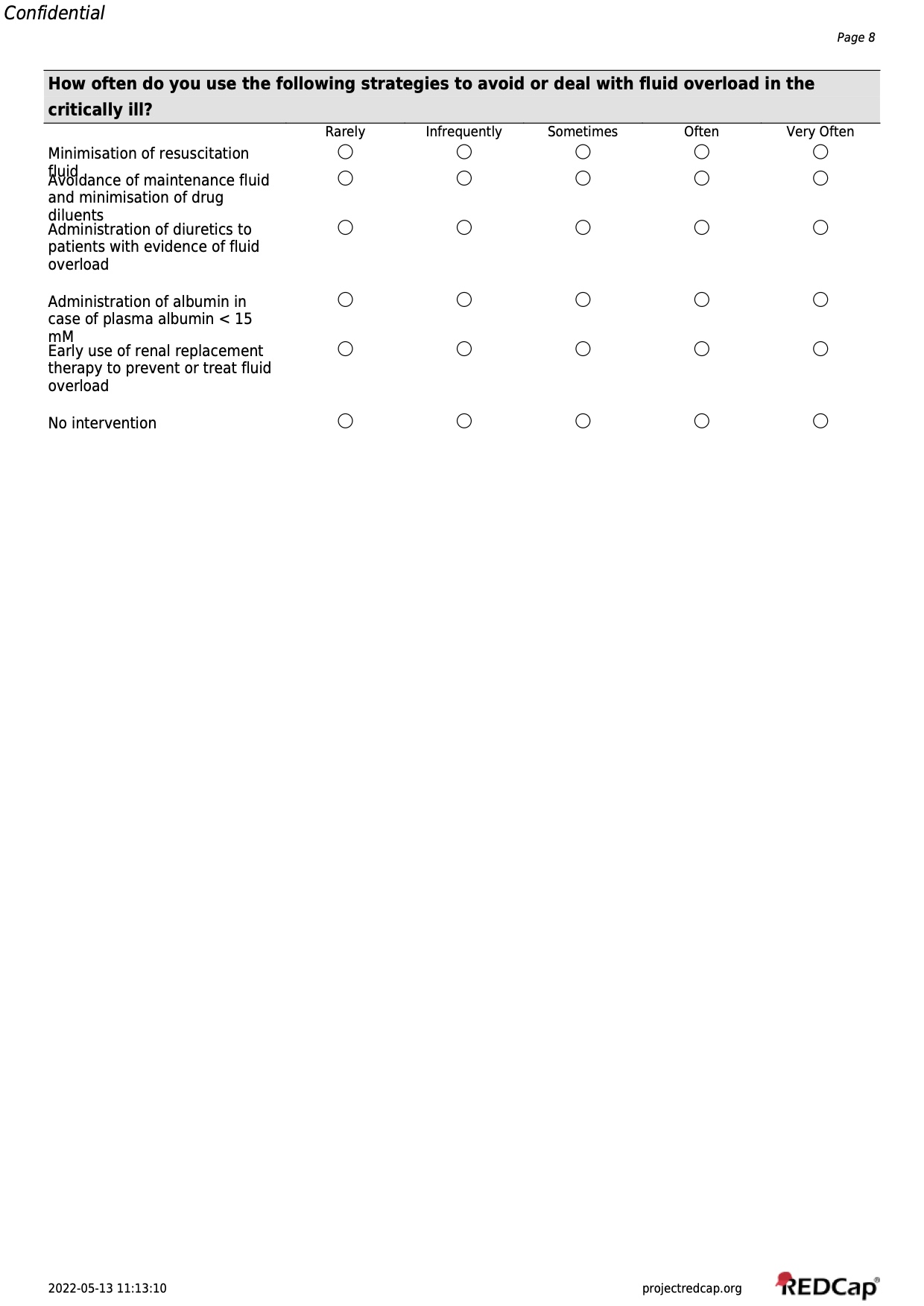

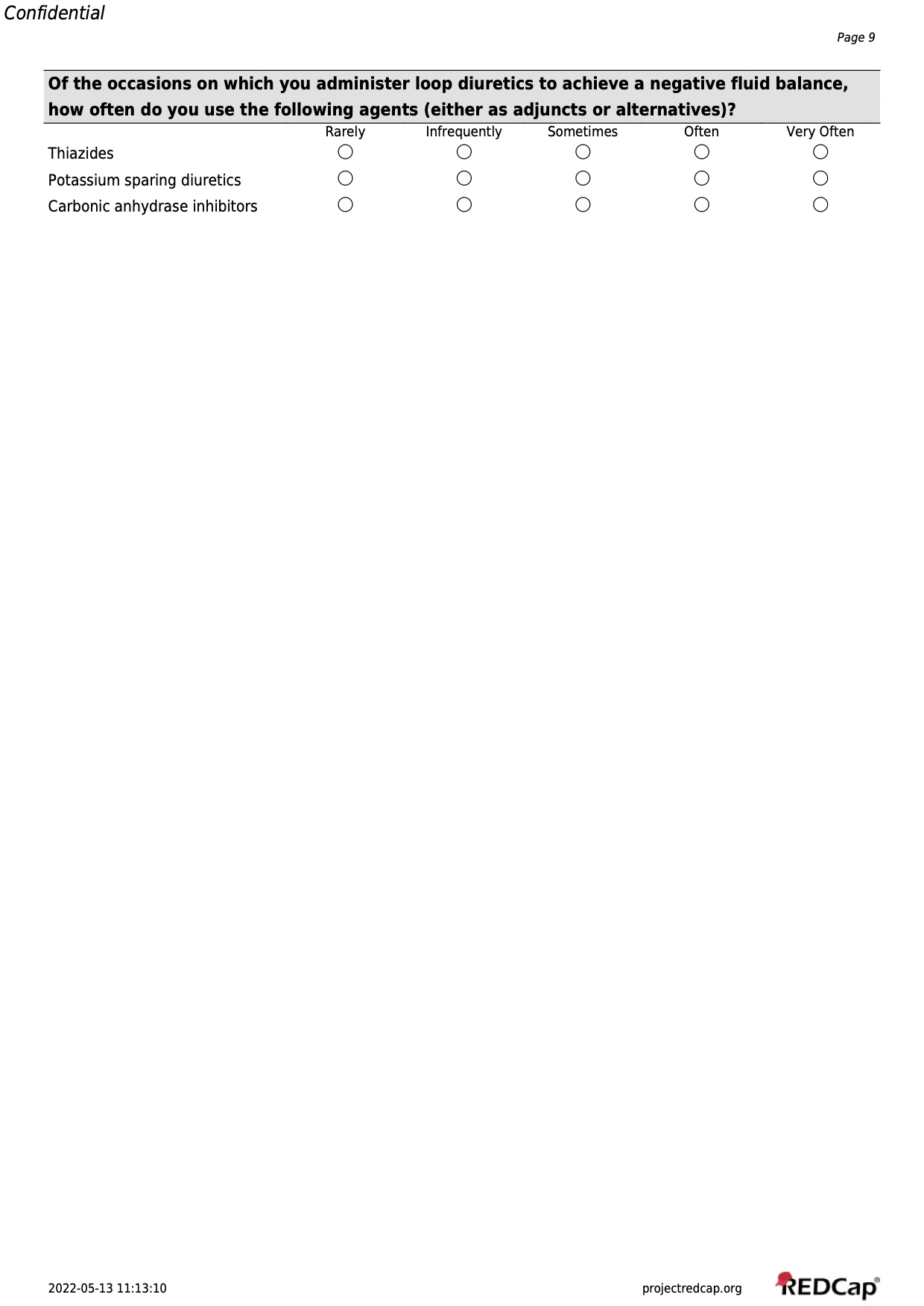

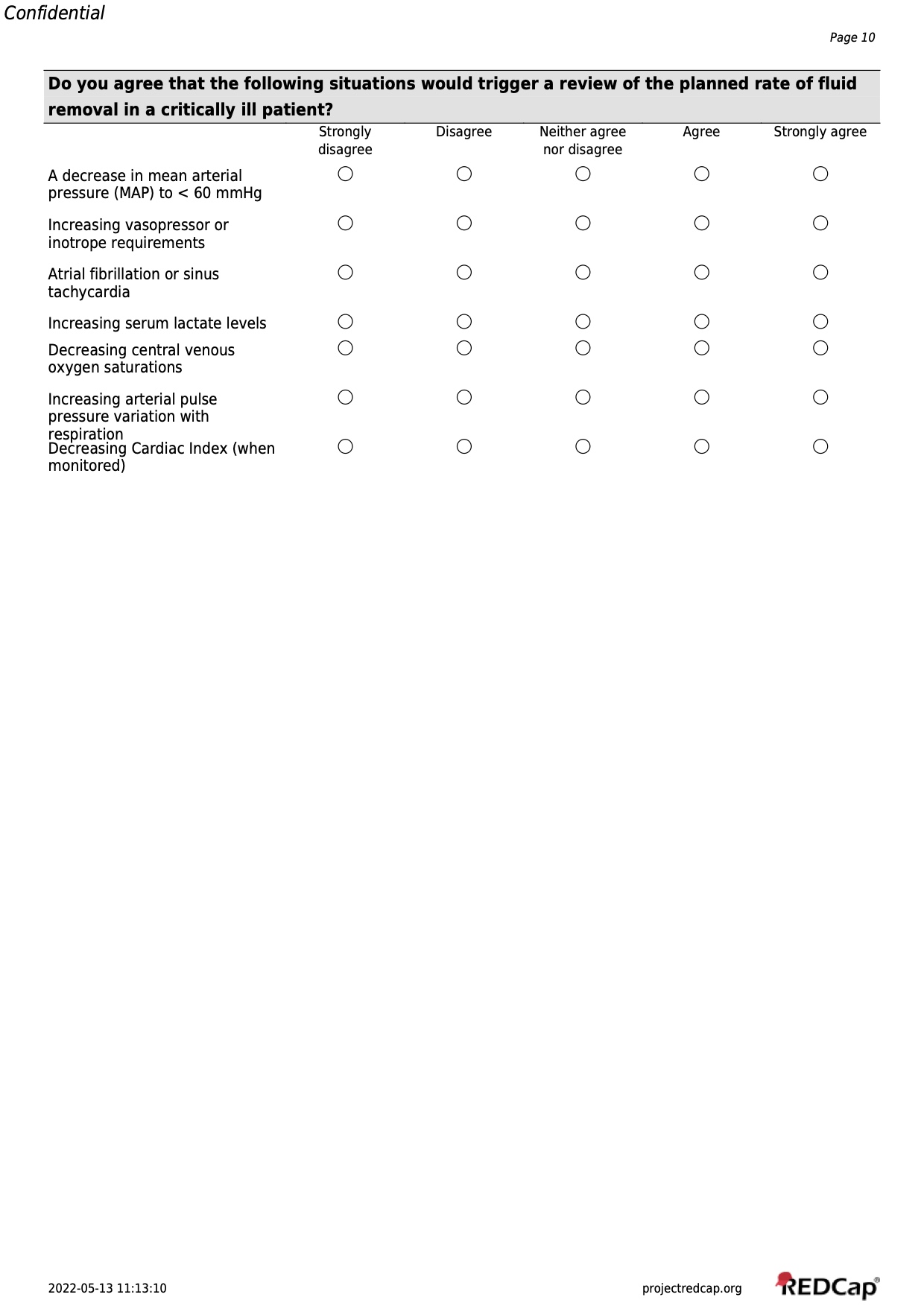

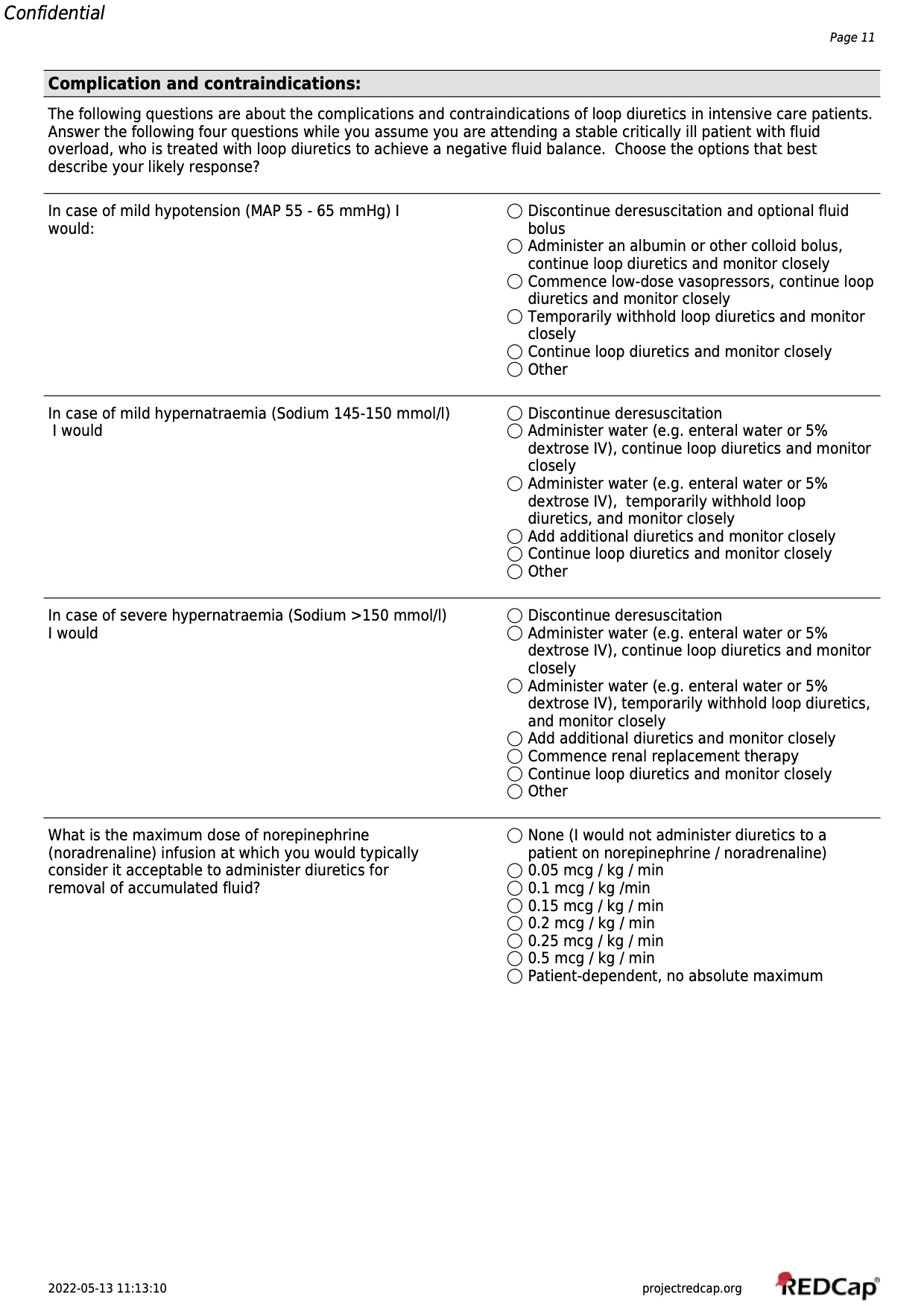


## **S3. Distribution survey to calculate response rate**

The distribution survey is attached as screenshots of the PDF document **
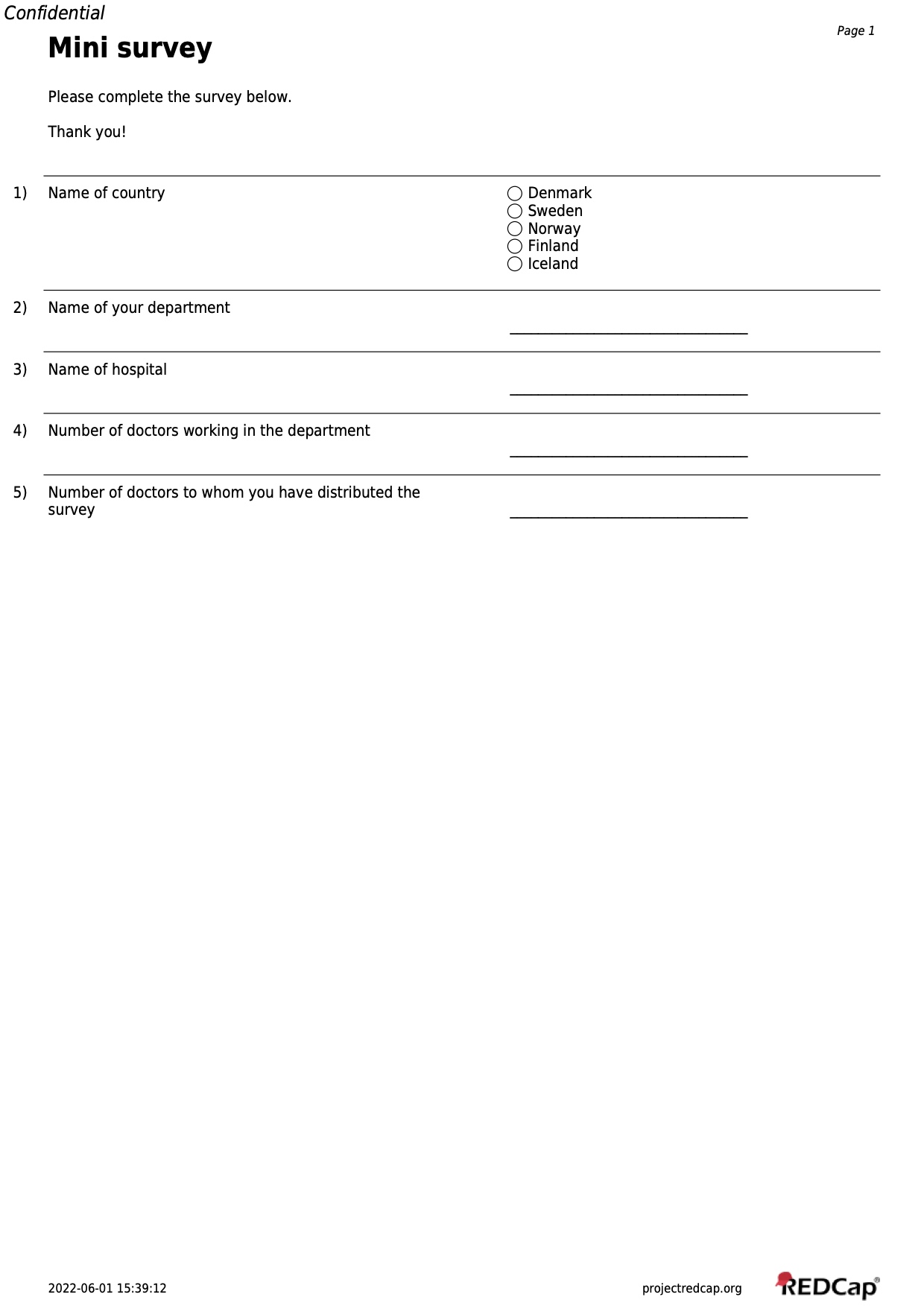
**

## **S4. Calculation of response rate:**

According to American Association of Public Opinion Research there are six definitions of response rate. (2)

We applied RR4:

$RR4=\frac{Complete surveys+ Incomplete surveys}{\left( Complete surveys+Incomplete surveys \right)+\left( refusal+noncontact+other \right)+e\left( unknown if eligible+unkonwn other \right)}$ (3)

Due to the lack of overview over the non-responders (refusals, non-contacts and other) we pooled all the non-respondents as unknown eligible respondents. The unknown eligible respondents are the difference between the number of emails sent and the number of survey respondents:

$$unknown eligble=3849-1066=2783$$

$$e is$$

the estimated proportion of cases of unknown eligibility that are eligible (3). There where 90 respondents in the survey that were excluded in the first question, because they did not work in the ICU, these are ineligible respondents. The sum of complete 564 and incomplete 412 cases are the eligible respondents. We hypothesized that the eligible proportion (e) would be the number of eligible respondents divided by the total number of individuals who commenced the survey 1066.

$$e=(\frac{Complete cases+Incomplete cases}{Number of individuals who commenced the survey}=0.915 )$$

$RR4=\frac{564+412}{\left( 564+412 \right)+0.915*\left( 2783 \right)}=0.277$ which means the response rate is 27.7%

## **S5. Calculation of non-response bias**

**What is non-response bias and how to estimate it:**

Non-response bias is the difference between the opinions of the respondents and the non-respondents. When creating a survey there can be a systematic reason for non-response, which could lead to results that are skewed. To assess the likelihood of bias we applied wave analysis. Wave analysis is in survey research a common method for estimating bias between respondents and non-respondents. In wave analysis non-response bias is assessed by comparing how initial respondents (first wave respondents) answer the questionnaire compared to late respondents (last wave respondents). Late respondents are more reluctant to answer the questionnaire and require reminders to respond to the survey. They are the group that resemble non-respondents most and are also called proxy non respondents (3).

**How we defined our first wave responders and our proxy non-responders:**
The true respondents/first wave respondents are defined as the 60 first Danish respondents who answered the questionnaire in the time interval between the 5.1.2022 to the 6.1.2022. These respondents received the invitation to participate in the survey and participated immediately. (Figure 1)

Second wave respondents answered the survey after one reminder and are therefore more willing to participate in the survey, than the third wave respondents and thus, not essential for calculating non-response bias. (Figure 1)

The third wave respondents /proxy nonrespondents are defined as 60 Danish respondents who answered the survey in the time interval 27.1.2022 and the 7.2.2022. In this time frame we only distributed the second reminder email. We hypothesize that the respondents in this time frame answer the survey because of the last reminder. (Figure 1)

We included only the Danish respondents in the calculation, because of a better overview of distribution of first, second and third email in Denmark compared to the four other Nordic countries. (Figure 1)


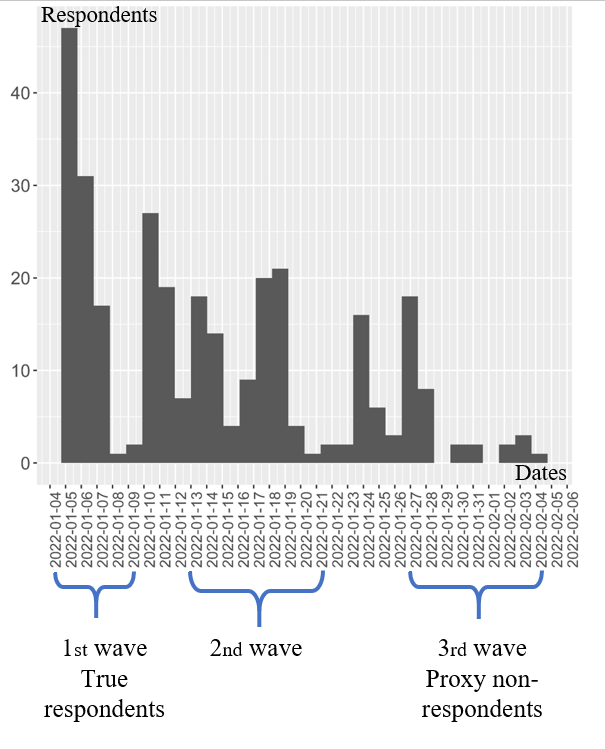


Graph over the Danish response rate from 5th of January to 6^th^ of February. Highlighting the periods where the survey was initially distributed (1^st^ wave) and when the first and second reminder was sent (2^nd^ and 3^rd^ wave)

**Calculations of non-response bias:**

We calculated the nonresponse bias for the attitudinal questions on fluid overload. These questions were Likert scale questions that can be translated on a scale on one through five. In this case 1= Strongly disagree, 2 = Disagree, 3 = Neither agree nor disagree, 4 = Agree and 5 = Strongly agree.
Thus, it is possible to calculate a mean answer for the first wave/true respondents and third wave respondents/ proxy non-respondents. (3)

$$Nonresponse bias=$$

$\left( proportion of nonrespondents \right) x \left[ (mean_{true respondents})-(mean_{Proxy nonrespondents}) \right]$(3)

$Proportion of nonresponders=\frac{Survey answers}{Survey distributed}=\frac{2783}{3849}=0.72$:

| **Question** | **Mean  for true respondents** | **Mean  for proxy  nonrespondents** | **Nonresponse bias** |
| --- | --- | --- | --- |
| **An inevitable consequence of appropriate fluid resuscitation in the presences of capillary leak** | 3.1 | 3.5 | -0.26 |
| **A modifiable consequence of fluid administration from multiple sources** | 4.0 | 4.1 | -0.1 |
| **A manifestation of sodium and water retention due to endocrine factors and acute kidney injury** | 3.4 | 3.5 | -0.1 |
| **An issue which will resolve spontaneously with resolution of underlying issue** | 3.1 | 2.9 | 0.17 |
| **A finding without clinical consequence** | 1.4 | 1.5 | -0.06 |
| **A modifiable source of morbidity** | 4.0 | 4.1 | -0.06 |

**Interpretation of the calculated non-response bias:**

The answers are interpreted on a five-point Likert scale, where two adjacent points represents e.g. “Agree” and “Strongly agree”. If they were close to 1 or larger this would mean the proxy nonrespondents would have an opinion that would represent an adjacent statement on the Likert scale and hence our results would be biased (3). In our case the non-response bias is between 0.06 and 0.26 which small numbers on the 5-point scale, demonstrating a low possibility of bias in the survey (see table 1).

## **S7. References**

1. Surveys ESICM. *Tools and questionnaires for applicants* (2021) https://www.esicm.org/research/surveys/

2. The American Association for Public Opinion Research. Standard Definitions: Final Dispositions of Case Codes and Outcome Rates for Surveys. 9th edition. *American Association for Public Opinion Research* (2016) https://www.aapor.org/Standards-Ethics/Standard-Definitions-(1).aspx [Accessed May 16, 2022]

3. Phillips AW, Reddy S, Durning SJ. Improving response rates and evaluating nonresponse bias in surveys: AMEE Guide No. 102. *Medical Teacher* (2016) 38:217–228. doi: 10.3109/0142159X.2015.1105945
